# Proportional estimation of finger movements from high-density surface electromyography

**DOI:** 10.1186/s12984-016-0172-3

**Published:** 2016-08-04

**Authors:** Nicolò Celadon, Strahinja Došen, Iris Binder, Paolo Ariano, Dario Farina

**Affiliations:** 1 Center for Sustainable Futures@PoliTo, Fondazione Istituto Italiano di Tecnologia, Torino, Italy; 2 Institute for Neurorehabilitation Systems, University Medical Center Göttingen, Göttingen, Germany; 3 Tyromotion GmbH, Graz, Austria

**Keywords:** Surface electromyography, High-Density electrodes, Machine learning, Human–machine interfaces, Rehabilitation robotics, Finger control, Hand rehabilitation

## Abstract

**Background:**

The importance to restore the hand function following an injury/disease of the nervous system led to the development of novel rehabilitation interventions. Surface electromyography can be used to create a user-driven control of a rehabilitation robot, in which the subject needs to engage actively, by using spared voluntary activation to trigger the assistance of the robot.

**Methods:**

The study investigated methods for the selective estimation of individual finger movements from high-density surface electromyographic signals (HD-sEMG) with minimal interference between movements of other fingers. Regression was evaluated in online and offline control tests with nine healthy subjects (per test) using a linear discriminant analysis classifier (LDA), a common spatial patterns proportional estimator (CSP-PE), and a thresholding (THR) algorithm. In all tests, the subjects performed an isometric force tracking task guided by a moving visual marker indicating the contraction type (flexion/extension), desired activation level and the finger that should be moved. The outcome measures were mean square error (nMSE) between the reference and generated trajectories normalized to the peak-to-peak value of the reference, the classification accuracy (CA), the mean amplitude of the false activations (MAFA) and, in the offline tests only, the Pearson correlation coefficient (PCORR).

**Results:**

The offline tests demonstrated that, for the reduced number of electrodes (≤24), the CSP-PE outperformed the LDA with higher precision of proportional estimation and less crosstalk between the movement classes (e.g., 8 electrodes, median MAFA ~ 0.6 vs. 1.1 %, median nMSE ~ 4.3 vs. 5.5 %). The LDA and the CSP-PE performed similarly in the online tests (median nMSE < 3.6 %, median MAFA < 0.7 %), but the CSP-PE provided a more stable performance across the tested conditions (less improvement between different sessions). Furthermore, THR, exploiting topographical information about the single finger activity from HD-sEMG, provided in many cases a regression accuracy similar to that of the pattern recognition techniques, but the performance was not consistent across subjects and fingers.

**Conclusions:**

The CSP-PE is a method of choice for selective individual finger control with the limited number of electrodes (<24), whereas for the higher resolution of the recording, either method (CPS-PA or LDA) can be used with a similar performance. Despite the abundance of detection points, the simple THR showed to be significantly worse compared to both pattern recognition/regression methods. Nevertheless, THR is a simple method to apply (no training), and it could still give satisfactory performance in some subjects and/or simpler scenarios (e.g., control of selected fingers). These conclusions are important for guiding future developments towards the clinical application of the methods for individual finger control in rehabilitation robotics.

## Background

The dexterity of human hand is the result of complex motor patterns that generate a coordinated response of multiple muscles placed intrinsically in the hand and in the forearm. The control signals to move each finger of the hand are generated in separate regions of the primary motor cortex (M1) [1], and are delivered to the muscles via the efferent pathways of the spinal cord and peripheral nervous system [2]. The neural commands elicit muscle electrical activity and a mechanical response. In recent years, it has been demonstrated that the intention to move the hand can be decoded using pattern recognition applied to recorded and processed electromyography (EMG) signals [3, 4]. This research was motivated by the importance to restore the hand function following amputation or an injury/disease of the nervous system, such as stroke. Most often, the aim was to detect less dexterous arm movements, such as the wrist rotations (e.g., pronation/supination) [5–7] and/or overall grasping patterns (e.g., palmar, lateral grip) [8, 9], whereas the classification and regression of finger movements has been less explored. Only recently, motivated by the development of modern dexterous hand prostheses [10] and hand exoskeletons [11–13], researchers started exploring the classification and regression of finger movements with the aim of establishing methods for intuitive control of these sophisticated systems, mimicking the dexterity of the human hand.

Most studies addressed the classification of individual finger movements (see Table 1). In this context, the aim was to predict the finger that moved but without proportional information (e.g., exerted force or position). Despite a good level of classification accuracy, generally higher than 90 % [14, 15], the discrete output of these pattern recognition algorithms led to a limited clinical applicability. In addition to discrete classification, continuous variables such as forces or positions can also be estimated from the EMG signals using regression. Regression algorithms have been applied under the main assumption that the EMG signal is related to the force generated by the muscle [16]. Since the force produced by muscles acting on a joint determines the position of the joint, the algorithms were trained to learn the mapping from EMG to force and/or position. Previous studies demonstrated that the hand kinematics can be estimated from surface EMG [6, 17–20]. For example, in [20], the authors proposed an innovative control strategy using a muscle activation model that parameterized the electro-mechanical delays (EMD). The study demonstrated good accuracy, estimating metacarpophalangeal (MCP), proximal interphalangeal (PIP) and the distal interphalangeal (DIP) finger joint with the mean correlation coefficient of 0.85 ± 0.07, 0.78 ± 0.06 and 0.73 ± 0.04, respectively. As pointed out in [21], the position control is effective only in the absence of interaction with objects. Since the functional applications include direct contact through grasping and manipulation, a force control is likely a more relevant solution. Recently, proportional control was investigated in the context of prediction of individual finger forces [22, 23], demonstrating that a non-linear incremental learning method could predict fingertip forces during flexion and extension with a correlation of ~0.9 between the estimated and measured forces.

**Table 1:** Journal papers on classification and regression of finger movements using electromyography

Ref.	Year	Classifier	Features	Finger moves	Subjects	Window (ms)	Electrodes	Accuracy	
[38]	2002	kNN	DFT, AR	F (T,I,M-R-L)	ND (4)	-	3	98 %	
[39]	2009	ANN	TD	F-E (T,I,M,R,L)	TR (1) ND (5)	200	32	90 %	Classification
[18]	2010	kNN	MAV	F (T,I,M-R-L)	TR (1)	250	16	86 %	
[68]	2010	EPM	TD	F (T,I,M,L,R)	ND (2)	-	4	>97 %	
[69]	2011	kNN	MAV	F (T,I,M,L,R)	TR (5) ND (5)	250	8	79 % (TR) 89 % (ND)	
[70]	2012	SVM, kNN	TD, AR	F (T,I,M,L,R)	ND (8)	250	2	90 %	
[14]	2012	LDA, SVM, GMM	TD, AR	F-E (T,I,M-R-L)	PS (12)	256	89	95 %	
[15]	2013	LDA, SVM	TD, AR	F-E (T,I,M-R-L)	ND (10) TR (6)	200 ms	12 11	98 % (ND) 90 % (TR)	
[41]	2014	KRLS	TD	F (T,I,M,R,L)	ND(40)	100–400	12	90 %	
[42]	2015	LDA	TD, AR	F (T,I,M)	ND(7)	250	5 (iEMG)	85 %	
[71]	2006	ANN	ENV	F (T,I,M,L,R)	TR(2)	-	8	(JA) Norm RMS error 8–20 %	Regression
[17]	2009	ANN	RMS	F-E (I)	ND (15)	100	1	(JA) RMS error 0.085 rad −0.163 rad	
[19]	2012	ANN	WL	F-E (T,I,M-R-L)	ND (5)	32	4	Norm RMS error 7–14 %	
[20]	2014	ANN, GP	EMD	F-E (T,I,M-R-L)	ND (10)	-	8	(JA) Mean CORR 0.85 ± 0.07 (MCP) 0.78 ± 0.06 (PIP) 0.73 ± 0.04 (DIP)	
[37]	2014	ANN	ENV	F-E (I,M,L,R)	ND (8)	-	14 – 16	(JA) R2 = 0.8	
[22]	2014	RR	RMS	F (T,I,M,R,L)	ND (10)	200	10	(FF) Norm RMS error 16 %	
[23]	2014	RR	ENV	F-E (I,M-R-L)	ND (10)	-	10	(FF) Norm RMS error 10–20 %	

*ANN* artificial neural network, *AR* autoregressive, *CORR* coefficient of correlation, *DFT* discrete Fourier transform, *E* extension, *EMD* electromechanical delay, *ENV* Envelope, *EPM* entropy probabilistic model, *F* flexion, *FF* fingertip forces, *GMM* gaussian mixture model, *GP* nonparametric gaussian process, *I* index finger, *JA* joint angles, *KRLS* kernel regularized least squares, *kNN K*-nearest neighbors, *L* little finger, *M* middle finger, *MAV* mean absolute value, *ND* nondisabled, *PS* post-stroke, *RA* regression accuracy, *R* ring finger, *RMS* root mean square, *RR* ridge regression, *SVM* support vector machine, *T* thumb, *TD* time domain, *TR* transradial amputee, *WL* waveform length

Recently, considerable attention has been devoted to investigating rehabilitation interventions which can facilitate the recovery of the sensory-motor functions impaired due to an injury/disease of the nervous system [24]. Numerous studies [25–28] demonstrated that the motor ability could be regained through a task-specific intensive practice. In this context, robotic rehabilitation is a promising method for the restoration and relearning of motor functions, since it can provide mass practice in well-controlled conditions [29]. Moreover, sEMG can be used to estimate the intention of the subject and operate the robot accordingly [12, 30–32]. This would create a user-driven control of a rehabilitation robot, in which the patient needs to provide a minimal activation to trigger and maintain the assistance. The benefit of this approach is that the subject is motivated to actively engage in therapy by recruiting his/her spared voluntary motor control, instead of passively relying on the robot to guide the movement [33]. Furthermore, the EMG control allows highly disabled patients who cannot produce detectable forces and/or motions, but can generate residual EMG, to participate early in the user-responsive therapy. More specifically, the context for the present work is the rehabilitation of selective finger movements using a specialized hand rehabilitation robot (Amadeo, Tyromotion GmbH, AT). Rather than aiming at the simultaneous control of multiple fingers to achieve functional movements (e.g., grasps) as required in prosthetics, the emphasis here is on the selective activation of individual fingers (i.e., one finger at a time) while reducing the simultaneous false co-activations. The motivation for this approach is to promote relearning of the selective motor control skills, which are heavily impaired in neurological patients (e.g., stroke [34]).

The present study advances the state of the art of individual finger control by investigating proportional estimation of fingertip forces during tasks that combined different force profiles, force levels and rates of change of force. Three different methods based on common spatial filtering (CSP-PE), linear discriminant analysis (LDA) and simple thresholding (THR) were applied to learn the mapping from High-Density sEMG (HD-sEMG) to finger activation; their performance were compared in offline/online tests and across different numbers of electrodes. To the best of our knowledge, such a comprehensive set of conditions was not considered before in the context of single finger classification and regression (see Table 1).

The focus of the present study was on the estimation of the finger forces using HD-sEMG to record the electrical activity of the extrinsic hand muscles during isometric finger flexion and extension. The thumb, however, has a specific anatomy and a functional behavior, with an additional degree-of-freedom (opposition) fully controlled through the intrinsic muscles. Consequently, the present study considered only the four long fingers. Nevertheless, the thumb activation could be estimated as well by placing additional electrodes over the intrinsic muscles. This could be accomplished using conventional bipolar electrodes, and therefore, this was not relevant for the present study.

HD-sEMG was selected since it provides a high resolution of sensing points, capturing the high-fidelity spatial and temporal patterns of muscle activity and revealing a topographical map of focal activation areas corresponding to individual muscles. The muscle heads moving individual fingers are located close to each other, within the relatively small volume of the forearm [35]. Therefore, HD-sEMG was chosen to selectively capture the individual muscle activity despite the significant spatial and temporal overlap. HD-sEMG has been used before to characterize the activity of the forearm muscles [36] [37]. However, the present study represents the first application where an HD-sEMG interface has been applied for individual finger movement classification and regression, investigating a comprehensive set of conditions that were not considered before. The high resolution of the recording (192 channels) was exploited to assess the robustness of the tested methods with respect to the reduction in the number of electrodes, providing important perspectives regarding the potential practical applications. Moreover, to the best of our knowledge, there are no studies presenting an online protocol of finger control based on HD-sEMG, evaluating three control methods: one direct (THR), and two based on pattern recognition (LDA and CSP-PE). Furthermore, the two methods, CSP-PE and THR, have not been considered before for the control of individual fingers. All the experiments were conducted using a commercial rehabilitation robot, mimicking closely a real clinical context. In conclusion, this study presents some important insights for guiding future developments towards the clinical application of the methods for individual finger control in rehabilitation robotics.

## Methods

**Fig. 1:**
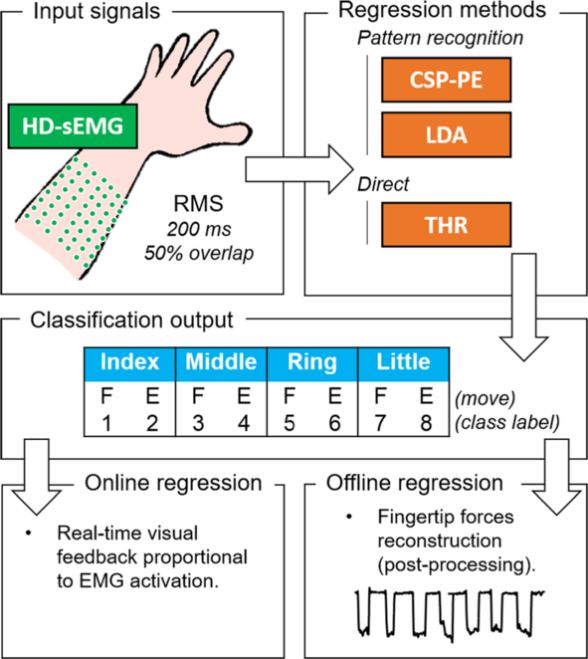
Outline of the experiments. HD-sEMG recordings were processed (root mean square, data windowing with overlap) and used as inputs for classification/regression to estimate the level of activation of individual fingers during flexion (F) and extension (E) movements. Two machine-learning approaches for myoelectric control, a standard benchmark (LDA) and a recently presented novel method (CSP-PE), as well as direct control via simple thresholding (THR) were assessed in the context of selective finger control. Both offline and online tests were performed. In offline tests, isometric forces of individual fingers were measured and predicted by applying the above-mentioned methods. During the online tests, the task for the subjects was to track the reference trajectories specifying the desired individual finger activation levels assessed using EMG normalized to maximum voluntary contraction. To this aim, the subjects controlled a visual marker, which was moving according to the finger activation levels predicted online using the selected estimation method

The aim of the study was to test different methods, for a dexterous finger control, estimating the intended level of activation of individual fingers (index, middle, ring and little) while minimizing the simultaneous unintended co-activations of other fingers during flexion and extension movements. Therefore, the task was to classify among 9 classes (four fingers x two movements and rest, see Fig. 1), with the simultaneous regression of the finger activation level within the selected class. For all three methods, the inputs were processed sEMG signals (feature vector), from the full set or subsets of electrodes, while the outputs were the estimated finger activation levels proportional to the exerted force. The regression was evaluated in the context of a linear discriminant analysis (LDA) classifier [38], a multi-class proportional estimator based on common spatial pattern (CSP-PE) [8] and a non-pattern recognition method based on a thresholds crossing (THR) [3], where the THR was applied only in the online experiment. The LDA was selected as a widely used method for movement classification and regression [39] (common benchmark). The CSP-PE was selected under the hypothesis that its mathematical properties would make the method especially effective in the context of selective finger activation, reducing the crosstalk between the estimated movements. The THR was chosen because it is a simple method, easy to understand, implement (no training) and apply even by a non-technical personal, and thereby convenient for prospective practical application in clinical settings. The hypothesis was that the THR could still perform well when used with the HD-sEMG interface due to its high resolution and ability to reveal focal areas of muscle activations. Summarizing, the methods were selected to compare: i) machine learning (CSP-PE and LDA) vs. direct (THR) control; and ii) a novel multiclass algorithm (CSP-PE) vs. the golden standard for the myoelectric control using pattern recognition (LDA). Their performance was compared in offline/online tests and across different subsets of electrodes, in order to assess how the nature of control (open vs. closed loop) and the resolution of the recording affect the performance, respectively. In the offline tests, the full experimental session could be devoted to data collection, leading to a comprehensive dataset enabling a thorough assessment of the methods across many conditions. The online tests included both training and assessment within a single session, and therefore only the selected conditions could be evaluated. As pointed in [40], in online control the user can exploit the visual feedback to adapt to the error mapping provided by the algorithms (closed-loop control), resulting in different performance when compared to offline estimation (open-loop control). Therefore, for an objective assessment, it is recommended to test both conditions. In offline tests, isometric forces of individual fingers were measured and offline predicted using the indicated estimation methods. During the online tests, the subjects activated the fingers to accomplish an online control task, while the selected method estimated the level of activation for each individual finger in real-time. The subjects received online visual feedback about the desired and estimated level of activation expressed as a percent of maximum voluntary contraction (MVC). In the online tests, the aim was to produce a control signal proportional to the exerted force, but the fingertip forces were not measured and directly estimated. Instead, the reference and estimated activation levels were calibrated according to the MVC of each subject, as measured by EMG (see section Online Experiment). To collect the training data, the subjects were asked to perform isometric tracking tasks, as in the previous studies [21, 23, 41, 42]. In offline tests, the reference trajectory was a predefined force profile expressed in Newtons, whereas in the online test, the reference profile was expressed as a percent of MVC.

### Experimental setup

**Fig. 2:**
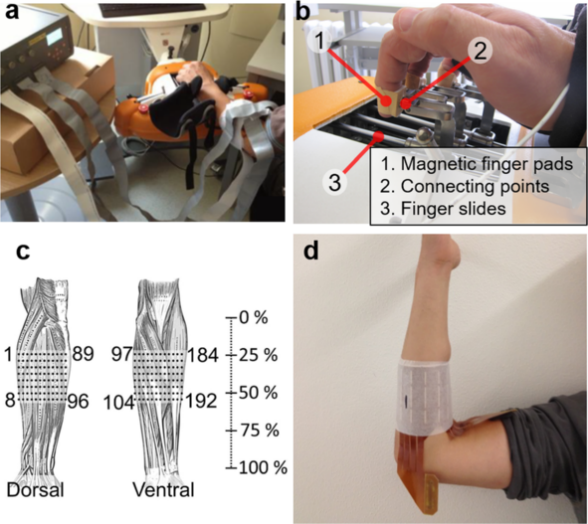
Experimental setup. **a** The subject’s arm positioned in the finger -hand rehabilitation robot (Amadeo, Tyromotion GmbH, AT), the multichannel EMG amplifier on the desk next to the subject, HD-sEMG electrode placed on the forearm and flat cables connecting the electrode to the amplifier. **b** Hand connection by means of magnetic pieces embedded in the finger pads. **c**-**d** Approximated position of the High-Density 192-channel electrode grid

The four long fingers of the dominant arm were attached to the finger slides of a robot specifically designed for the hand rehabilitation in stroke patients (Amadeo, Tyromotion GmbH, AT) as indicated in Fig. 2-b. Magnetic pieces were embedded in the ergonomic finger pads that were secured to each finger tip using medical tape. The pads were then positined on the respective magnetic connection point of each finger slide. Magnetic force was enough to keep the fingers in position during all experimental conditions in healthy subjects (see Fig. 2-b). The slides were driven individually using dedicated linear motors instrumented with position and force sensors. The position of the subject wrist and the slides was adjusted for each subject individually so that the PIP joints were flexed at approximately 90° while the DIP joints of all fingers were fully extended (180°). Such an “orthogonal” setup allowed for an optimal transmission of forces between the fingers and the robot guides and sensors. After setting up the hand configuration, the slides were kept in stationary positions during the rest of the experimental session, measuring individual finger forces during isometric contractions. The sensor range for finger extension and flexion was ± 20 N. The forces were recorded only during the offline experiment. The signals were sampled at 10 Hz, internally by the robot controller, sent to the host computer via TCP/IP and shown on a computer screen as feedback to the subject.

The EMG signals were recorded using a High-Density 192-channel electrode grid (ELSCH064NM 3–3, OT Bioelettronica, 8x24 channels, 10 mm inter-electrode-distance, 8 × 24 cm) in a monopolar configuration placed on the dominant arm. The forearm length was measured in each subject using a measuring tape. The electrode array was positioned 6.4 ± 0.4 cm (25 % of the forearm length) from the elbow crease (Fig. 2c and d), covering 8 cm of the forearm longitudinally and 24 cm circumferentially. The electrode configuration allowed acquiring the sEMG activity of distal and proximal muscles such as the *flexor digitorum superficialis* and *extrensor digitorium*. The EMG signals were recorded using a multichannel electrophysiological amplifier (EMG-USB2, OT Bioelettronica, IT) connected to the host computer via a USB port. The gain was set to 500, the signals were band-pass filtered (eight order analog Bessel filter, bandwidth 10-750 Hz), sampled at 2048 Hz and digitally converted (12 bit A/D converter, 5 V dynamic range) with a resolution per least significant bit of 2.44 μV. The reference electrode was a ground strip placed at the distal end of the forearm, just next to the wrist joint.

### Regression methods

The present study aimed at comparing three different proportional controllers (LDA, CSP-PE and THR) as they are applied to decode the activation of individual fingers from HD-sEMG patterns. The inputs were the processed EMG signals, and the regression methods outputted the estimated levels of finger activation proportional to the exerted force. The raw EMG data were segmented into a series of overlapping data analysis windows. The window length of 200 ms with 50 % overlap was selected since it represents a good trade-off between classification accuracy (CA) and controller delay [43]. The Root Mean Square (RMS) was computed over the data window and used as an input for the classification/regression, since it is a time domain feature related to the force exerted by the muscle [43]. Therefore, a class decision was produced for each data analysis window (every 100 ms), where the input for the regression was a vector of RMS values (one per electrode) computed over the 200-ms data window [44].

The LDA classification represents one of the most popular pattern recognition methods for myoelectric control. In summary, it models the distribution of the data within each class using a Gaussian distribution, where the means are estimated for each class individually and the covariance matrix is computed over the pooled data (shared covariance). The classification is performed by computing the class posterior probability (Bayes rule), which in the case of a shared covariance reduces to evaluating the linear discriminant functions separating the classes [45]. As demonstrated by several studies, this simple and fast method performed similarly to or even better than the other, more complex non-linear pattern recognition methods for time-domain features [46, 47]. Since the LDA classifier has become the golden standard for the pattern recognition of EMG signals, it was selected as common benchmark. In this study once a class decision was taken, a proportional control value was extracted in a different way depending on the experiment. In the offline test, this value was extracted as an approximation of the recorded fingertip forces obtained by linear regression. In the online test, as the mean of the RMS values from a subset of channels related to the specific class, scaled to a percentage of MVC for the detected movement class.

Common Spatial Patterns (CSP) is a semi-supervised algorithm to determine a filter whose output has maximal and minimal variance when the multichannel input data come from the first and second class, respectively. Therefore, the filter maximizes the separation of the two classes based on the variance of the filter output signal. Commonly, CSP is used as a spatial filter for raw signals of two distinct classes, but there are several options for the extension to multi-class problems [48]. The method has been originally used in brain-computer interfacing as a spatial filter for data preprocessing [49–51]. However, it has been recently adapted and tested for classification and regression in myoelectric control of prostheses, with promising results [8]. In this study, we applied CSP as a proportional estimator (CSP-PE) in one vs. one configuration between all possible class pairs, as presented in [8]. The CSP-PE has been selected since this is a novel method for myoelectric control with unique mathematical properties. Namely, the method aims at maximizing the contrast between classes and thereby minimizing the false co-activations. This indicates that it could be especially effective in the context of selective finger activation, which is the aim of the present work. In summary, the first step in the application of the CSP-PE was to determine the CSP filters for each pair of classes. The output of each filter was therefore tuned to maximize the response for the input data (feature vector) coming from one class, and respond minimally to the data from the other class. In the second step, the outputs of all the pairwise-class filters were fused in post-processing to estimate the class corresponding to the input data. Therefore, the activation of the class was determined by taking into account its relative contrast with respect to all the other classes. The selected class is the one that wins the competition, and the estimated activation reflects the uncertainty of this decision. For example, if the class loses at least one competition, the activation will be penalized leading to a small value. The raw outputs (activation levels) were scaled to yield a maximum value for 100 % of MVC. The method, including the original CSP formulation as well as the novel steps for CSP-based classification and regression, are given in detail in [8].

The THR is a simple method that involves direct control of each individual finger by identifying the focal areas of activity within the HD-sEMG interface. The THR was chosen because it is a simple method, easy to understand, implement (no training) and apply even by a non-technical personal, and thereby convenient for prospective practical application in clinical settings. The finger movement was detected if the activity at the selected subset of channels was above the predetermined threshold. THR was applied only during the online tests and a set of relevant channels was selected for each class (finger × movement) by the experimenter based on the visual observation of the EMG activity generated during the respective movements. The calibration trials were used to adjust the thresholds for each set of channels maximizing the correct classification. A class was recognized when the mean of the RMS values over the associated channel set crossed the threshold, and the RMS was higher than for the other classes. The proportional control value was the mean of the RMS values for the set of channels, scaled to a percentage of the MVC of each movement.

### Offline experiment

Nine healthy subjects (age between 26 and 41 years) participated in the experiments, which were approved by the Ethical Committee of the University Medical Center Göttingen (UMG). Before starting the tests, the subjects signed an informed consent form. The experimental session lasted approximately 1.5 h.

#### Experimental protocol

Subjects performed cyclical isometric contractions activating individual fingers in the direction of flexion and extension, as specified by the reference force profile presented on the screen. The marker indicating the currently generated force was displayed on the computer screen as the feedback for the subject. The subjects were instructed to activate the fingers selectively by minimizing simultaneous activation of other fingers. Before the beginning of the training session, the subjects were allowed to familiarize with the experimental setup and tasks. Twelve different tasks were carried out in random order with regard to the finger and each task was repeated 10 times in succession. The tasks combined force profiles (square with 50 % duty cycle and triangular), force levels (33 and 66 % MVC) and two execution speeds for each force profile (see Table 2), evaluating thereby how well the methods estimated the force during gradual increase/decrease (triangles) and level holding (squares) for different rates of change and peak forces. There were trials comprising only flexions, only extensions or both contraction types.

**Table 2:** Tasks included in the offline assessment. The tasks combined square (S) and triangular (T) force profile, force levels (33 and 66 % MVC) of flexion (F) and extension (E), and two execution speed for each force profile

N	Movement	Profile	% MVC	Cycle Length (sec)	
1	F	S	33	6	Training
2	F	S	66	12	
3	F	T	66	8	
4	F	T	66	4	
5	E	S	33	6	
6	E	S	66	12	
7	E	T	66	8	
8	E	T	66	4	
9	F-E	S	33	6	Testing
10	F-E	S	66	12	
11	F-E	T	66	8	
12	F-E	T	66	4	

#### Signal processing

Force signals were analyzed in order to extract segments of sEMG activity associated with specific muscular contraction (flexion or extension), level of force and duration. The processed EMG signals (windowing, RMS) had an effective sampling frequency of 10 Hz (one value per 100 ms) synchronized with the force recordings, performed at the same sampling rate. For each task, the force signal measured from the finger involved in the task was used to identify the flexion/extension force cycles, in order to leave out from the analysis the signal portion without the link to specific movements. A force cycle was defined by detecting when the generated finger force crossed the predefined threshold, which was set to 10 % of the maximum force exerted with that finger across all tasks, indicating the start of the contraction. When the force returned to a subthreshold level (<10 %), this denoted the end of the contraction. Occasional atypical contractions (outliers) were identified on the basis of the force cycle length and the force range. A regression line was estimated from the midpoints of the plateaus in the case of square profile and from the vertices of the triangle profiles. The mean and the standard deviation of the cycle length, and the Mean Squared Error (MSE) between the generated force during plateau and the regression line were calculated. Force cycles that were longer or shorter (*T*_*outlier*_) than 2.58 times the standard deviation from the mean duration (*T*_*mean*_) (see Eq. 1) or for which the force error (*e*_*outlier*_) was higher than 1.96 times the MSE from the regression line were considered as outliers (see Eq. 2) and excluded from the analysis. The confidence levels (1.96 × std and 2.58 × std) were chosen in order to enforce conservative outlier detection. The interval of ±1.96 × std corresponds to the standard 95 % confidence interval. The detected outliers were also confirmed by a visual check.

1 $$ {T}_{outlier}<\left(2.58\ast std\right)-{T}_{mean} \vee {T}_{outlier}>\left(2.58\ast std\right)+{T}_{mean} $$

2 $$ \left|{e}_{outlier}\right| > 1.96\ast MSE $$

#### Data analysis

We investigated the performance of the algorithms with full (192) and reduced number of channels (96, 48, 24, 16, 12, 10, 8, 6 and 4) where the channels were selected in the form of regular grids (see Fig. 6-e). The latter was chosen having in mind the future practical application of the methods, which should ideally, for the sake of simplicity, rely on the regular placement independent of the anatomy or the activity hot spots. The recorded data for each subject was split into training and testing sets (approximately 70 and 30 % of the whole dataset respectively, see Table 2): the tasks with only flexion or extension contractions were assigned to the training set (Table 2, Task 1–8), while the tasks with both flexion and extension were selected for the testing (Table 2, Task 9–12). The performance measures were common for online and offline tests and are described below (see section Performance measures).

### Online experiment

Nine healthy male subjects (age between 23 and 38 years) were recruited for the online algorithm evaluation. Each subject signed an informed consent before commencing the experiment, which was approved by the Ethical Committee of the University Medical Center Göttingen (UMG). The same test was performed twice on consecutive days in order to evaluate the effect of training. The experimental session lasted maximum 2 h.

#### Training data collection

**Fig. 3:**
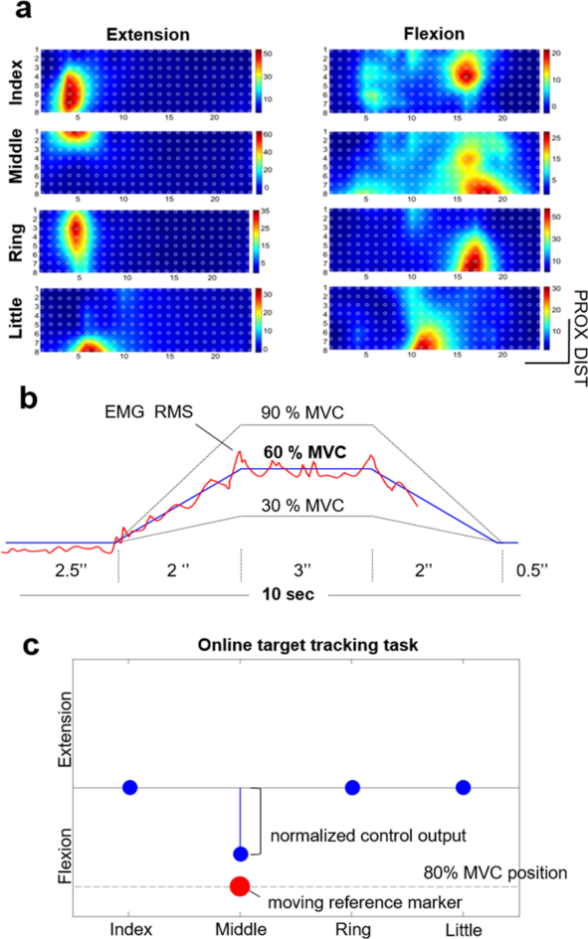
Three phases of the online experiment. **a** sEMG Root Mean Square (RMS) maps calculated over a 200-ms data window of (1 RMS sample). The RMS for each channel (*white circle*) was color coded as indicated by the color legend (μV), and the pixels between the channels were obtained by interpolation. The figure shows one representative subject performing isometric sustained contractions of individual fingers in the direction of flexion or extension. The fingers produce characteristic and spatially localized, but partly overlapping, EMG responses. **b** Training data collection. The subjects were asked to reproduce trapezoidal reference activation profiles (i.e., gradual increase, plateau, gradual decrease) with plateau at 30, 60 and 90 % of MVC of the respective finger. The *red* and *blue lines* depict the generated and reference activation levels from an example tracking trial at 60 %, respectively. **c** Online test where the subjects controlled 4 visual markers (*blue circles*), each associated with the activation of one finger, as indicated by the horizontal axis. The vertical position of the marker was set by the output of the tested control method (LDA, CSP-PE and THR) computing the estimated finger activation level. The subjects therefore proportionally controlled the vertical position of the marker by increasing or decreasing the finger force in the direction of flexion (marker moving downward) or extension (marker moving upward). The task for the subject was to activate the fingers, one at a time, tracking online the reference marker (*red sphere*) moving along vertical direction and representing the desired finger activation level

The subjects were asked to perform sustained isometric contractions activating selectively one of the four fingers in the direction of flexion or extension. For each movement, the experimenter selected groups of electrodes (from 4 to 6) in which the maximum activity was observed (Fig. 3-a) and measured the MVC as the maximum of processed sEMG (RMS, 200 ms window, 50 % overlap) over the specific electrode subsets. After the electrode subsets were chosen, the subjects were asked to reproduce the reference activation profile after receiving a visual cue indicating the finger and the contraction type (flexion or extension). Auditory “icons” (sound beeps) were used in addition to the visual cues. The reference profiles were trapezoidal (i.e., gradual increase, plateau, gradual decrease) with the plateaus of 30, 60 and 90 % MVC of the respective finger (Fig. 3-b). The current muscle activation level generated by the subject was indicated by a cursor moving with the constant velocity in the horizontal direction. The vertical coordinate of the cursor was equal to the mean RMS of the electrode subset corresponding to the current class (finger × contraction type + rest). Each contraction lasted for 7 s (2 s rise time, 3 s plateau, 2 s fall time) with 5-s rest intervals in-between and it was repeated 3 times (30, 60 and 90 % MVC), resulting in 27 contractions in a single run. If necessary, specific contractions could be repeated. For the training of both machine learning methods the same data set was used and dynamic movement phases were not excluded [52].

#### Online test

After the training, a test with online control was performed. The subject controlled 4 visual markers (blue circle in Fig. 3-c), each associated with the activation of one finger. The subjects now actively and proportionally controlled the vertical position of the control marker by increasing or decreasing the finger force in the direction of flexion (marker moving downward) or extension (marker moving upward). The vertical position of the marker (blue circle in Fig. 3-c) was determined by a normalized output of the tested control method (CSP-PE, LDA and THR), as explained in section Regression Methods. To quantify the control performance, the subjects performed online target tracking tasks, in which the subject tracked a moving reference marker (red circle in Fig. 3-c) by activating appropriate finger at the appropriate level. The aim was to maintain the smaller blue marker (Fig, 3-c), indicating the estimated activation level, within the larger red circle (Fig. 3-c), representing the desired activation. Whenever the controlled marker was within the reference circle, the color of the reference would turn into green. The task comprised 16 individual finger activations (4 for each finger) in which the reference marker moved vertically from 0 % MCV to 80 % MVC position, rested on the 80 % MVC level, and then returned back to the 0 % MVC value. The upward and downward movements of the reference marker lasted for 4 (slow ramp) or 2 (fast ramp) seconds, respectively, and the marker stayed at the plateau level for 3 s. Each finger was therefore activated two times in flexion and two times in extension and for both contractions one cycle was slow and the other fast. The fingers (reference markers) were activated in a random order. The test was repeated for each control method, also in a random order to avoid that the learning across methods influences the performance. The subjects were blinded as to which algorithm was under test in each session.

### Performance measures

The estimated fingertip forces in the offline experiment and muscle activation levels in the online experiment were low-pass filtered using a moving average filter applied to 5 successive samples starting at each sample of the original signal. The Pearson correlation coefficient (PCORR) between the estimated and reference forces was computed to quantify the similarity in the signal shapes, and the mean square error normalized (nMSE) by the peak-to-peak value of reference force profile was calculated to assess the difference in signal amplitudes [22]. As shown in Results section, the trends revealed by the two outcome measures, nMSE and PCORR, were equivalent over a comprehensive dataset collected and analyzed in the offline experiments. Therefore, the quality of the online tracking was evaluated by computing the nMSE only. In both the experiments, this analysis was performed for each finger during the segments of the reference trajectory in which that specific finger was supposed to be active (target finger in the task). The corresponding segments were named active phases. In order to evaluate the amount of false finger activations (i.e., finger estimated to be active when it should have been relaxed), the mean amplitude of the false activations (MAFA) outside of the respective active phases was calculated. The segments of the reference trajectory in which the finger was not supposed to be activated were named silent phases. The CA was evaluated calculating the overall success rate as the trace of the confusion matrix, divided by the total number of classified instances. Finally, the selectivity and specificity of the classifiers were calculated in one vs all configuration, where the median success rate of all the classes was used to compare the performance of the two methods for different electrode subsets.

### Statistical evaluation

The Kolmogorov-Smirnov test determined that the data were not normally distributed. Therefore, the data were statistically analyzed using non-parametric tests. To assess the statistically significant difference at the group level, the Friedman test was applied. If the Friedman test determined the difference, the conditions were compared pairwise using the Wilcoxon signed-rank tests with Bonferroni correction. A level of *p* < 0.05 was selected as the threshold for the statistical significance. In the offline experiment, the factors were number of channels (96, 48, 24, 16, 12, 10, 8, 6 and 4), and method (CSP-PE and LDA). In the online experiment, the factors were finger movements (IF, IE, MF, ME, RF, RE, LF, LE), and method (CSP-PE, LDA and THR). Bartlett multiple-sample test for equal variances was applied to determine statistically significant difference in dispersions within the conditions overall, followed by Ansari-Bradley two-sample test with Bonferroni correction for pairwise comparisons of the force variability between the conditions.

## Results

### Offline finger force prediction

**Fig. 4:**
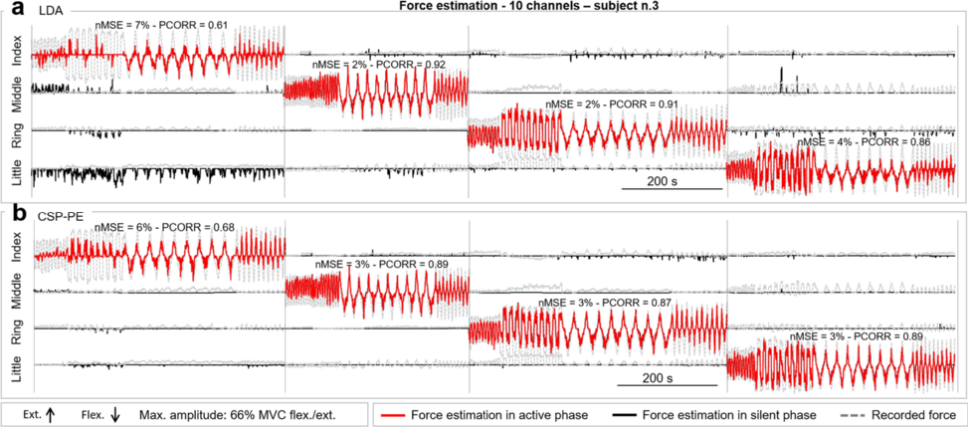
Representative results from one subject during offline experiment. Figure shows the individual finger force estimation when applying the LDA (**a**) and the CSP-PE (**b**) using a subset of 10 sEMG channels selected as a regular grid as input for the regression. The task for the subject was to activate the fingers individually and selectively, one at a time. The dashed *gray line* is the recorded finger force. The continuous *red line* is the estimated force for the finger that was supposed to be active in the task (active phase), while the continuous *black line* is the estimated force for the fingers that should have been relaxed (silent phase, false activation). The vertical lines delineate the active phases for different fingers. The quality of tracking is similar for the two methods, in this specific example, but the CSP-PE generated less false activations. For example, compare the false activation of the little finger in LDA vs. CSP-PE during the active phase of the index finger

Figure 4 illustrates the finger force estimation in one representative subject, when applying the LDA (Fig. 4-a) and the CSP-PE (Fig. 4-b) to a subset of 10 sEMG channels selected as a regular grid (see Fig. 6-e). During active phases (red line), both regression methods successfully tracked the force trajectories of different shapes and rates of change, i.e., triangles with faster/slower slopes and squares with longer/shorter plateaus. In this specific configuration, with only 10 electrodes, the force profile for the index finger was estimated with the lowest accuracy, and the estimation was better with the CSP-PE (nMSE = 6 %) than the LDA (nMSE = 7 %). During the silent phases, the LDA generated false activations that were more frequent and with the higher amplitudes compared to the CSP-PE. For example, the LDA falsely estimated that the little finger was activated substantially and consistently throughout the active phase of the index finger (Fig. 4-a).

**Fig. 5:**
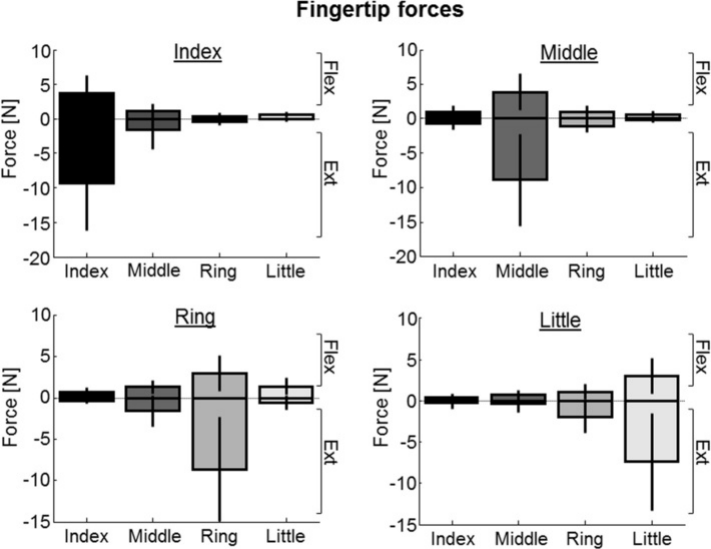
Fingertip forces (mean ± standard deviation) recorded during the offline experiment across different finger tasks. The subjects could not generate totally isolated movements of individual fingers, due to the natural passive coupling of the fingers

The recorded forces (gray lines) during the silent phases showed that the subject exerted a small pressure on the force sensors also outside the active periods. This was because the subject could not generate perfectly isolated activations of individual fingers, due to the natural passive coupling [53]. Group data are represented in Fig. 5, depicting the fingertip forces (mean ± standard deviation) recorded from all subjects across different finger tasks. The coupling between the fingers is evident, and the amount of force decreases for the fingers further away from the activated one. Nevertheless, the force in the active finger was several times higher from all the others, and the difference was statistically significant, demonstrating the selective activation.

**Fig. 6:**
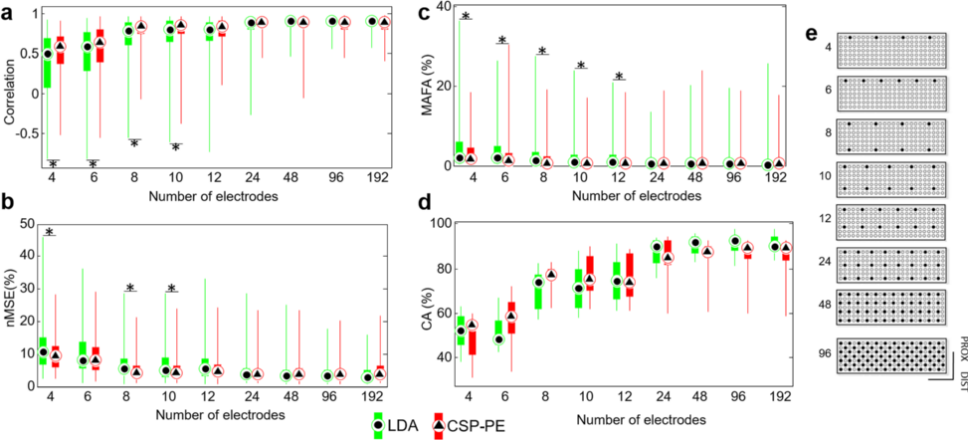
Summary results (median and interquartile range (25–75 %)) of the offline experiment. Different performance indices were evaluated: **a**) the coefficient of correlation and **b**) the normalized mean square error between the measured and estimated force signals during active phases, **c**) the mean amplitude of the false activations during silent phases and **d**) the classification success rate. Note that for the reduced electrode sets (<24), the CSP-PE outperformed the LDA in the accuracy of force estimation as well as in the suppression of false activations. Figure **e**) reports the different set of electrodes selected from the 192 HD-sEMG matrix. *p < 0.01

Figure 6 shows the summary results for the quality of estimation (median and interquartile range - IQR 25–75 %) using different number of electrodes. For both methods, the performance initially increased with more electrodes, i.e., the regression and classification become more accurate and the false activations less pronounced. Eventually, however, all outcome measures reached a plateau (*N* ≥ 24), after which there was no substantial further improvement. In terms of regression accuracy (Fig. 6a-b), the outcome measures (PCORR and nMSE) were consistent. For less than 24 electrodes, the CSP-PE outperformed the LDA (*p* ≪ 0.001), but for the higher number of electrodes the performance was similar with no statistically significant differences. For example, with 96 channels the nMSE values obtained with the LDA and the CSP-PE were virtually identical, i.e., (median and IQR 25–75 %) 3.5 % (2–5.1 %) and 3.6 % (2.1–5.3 %) (*p =* 1), respectively. When using more than 24 electrodes, PCORR was approximately 0.88 (median) for the CSP-PE and 0.90 (median) for the LDA. Similarly, the MAFA depended on the number of electrodes and the regression method (*χ*^2^ = 1773.30, *p* ≪0.001). For less than 24 channels, the MAFA was consistently lower for the CSP-PE compared to LDA (*p* < 0.001); when increasing the number of electrodes, the MAFA saturated to values lower than 0.5 % (median) with both of the methods.

Similar to the previous results, the CA (Fig. 6-d) increased with the number of electrodes (*χ*^2^ = 127.95, *p* ≪0.001), but in this case there was no statistically significant difference between the methods. The CA also saturated at 24 electrodes and the overall highest value (median and IQR 25–75 %) was reached using the LDA, i.e., 92 % (88.2–94.5 %). As described in Table 3, the specificity of the classifiers was highly affected by the number of channels: e.g., with only 4 channels the sensitivity of the CSP-PE and the LDA was still approximately 85 % (median), but the specificity dropped to 55 % (median). In conditions where the number of channels (*N* ≥ 24) saturated the performance of the classifiers, the sensitivity was approximately 96 % (median) and the specificity approximately 90 % (median). Summarizing, the two classifiers were more sensitive than specific, and there were no statistically significant differences between the classifiers (LDA vs. CSP-PE) with respect to both outcome measures.

**Table 3:** Percentage of selectivity and specificity across different electrode subset extracted from the HD-sEMG matrix. The values are reported as median and interquartile range (25–75 %)

CH	Sensitivity (%)		Specificity (%)	
	CSP-PE Median and IQR (25–75 %)	LDA Median and IQR (25–75 %)	CSP-PE Median and IQR (25–75 %)	LDA Median and IQR (25–75 %)
4	85 (81–86)	84 (82–87)	55 (45–61)	54 (47–58)
6	87 (84–89)	84 (82–86)	59 (51–72)	52 (48–58)
8	93 (92–93)	92 (87–93)	81 (77–83)	75 (66–80)
10	92 (91–95)	91 (88–93)	79 (74–87)	74 (69–81)
12	92 (89–96)	92 (89–94)	81 (70–88)	77 (71–84)
24	95 (94–98)	97 (94–98)	86 (84–93)	91 (85–94)
48	96 (95–97)	97 (96–98)	90 (86–92)	92 (88–95)
96	96 (95–97)	97 (96–98)	90 (87–92)	92 (89–94)
192	96 (95–97)	97 (96–98)	90 (87–92)	91 (90–94)

### Online control performance

There was a trend indicating an increase in performance across the two sessions, since the median values of the outcome measures improved, i.e., the nMSE and MAFA decreased with statistically significant differences (respectively, *χ*^2^ = 221.2 *p* ≪0.001 and *χ*^2^ = 163.7 *p* ≪0.001). The effect of training was least expressed when using the CSP-PE, which was characterized with a high level of accuracy in both sessions. For example, the median nMSE decreased for only ~1 % for CSP-PE (*p* ≪0.001) in the second session compared to the first, while in the case of LDA and THR, the median improvement in tracking accuracy was approximately 6 % between the two sessions (*p* ≪0.001). The results reported in this section refer to the second session of the experiment.

**Fig. 7:**
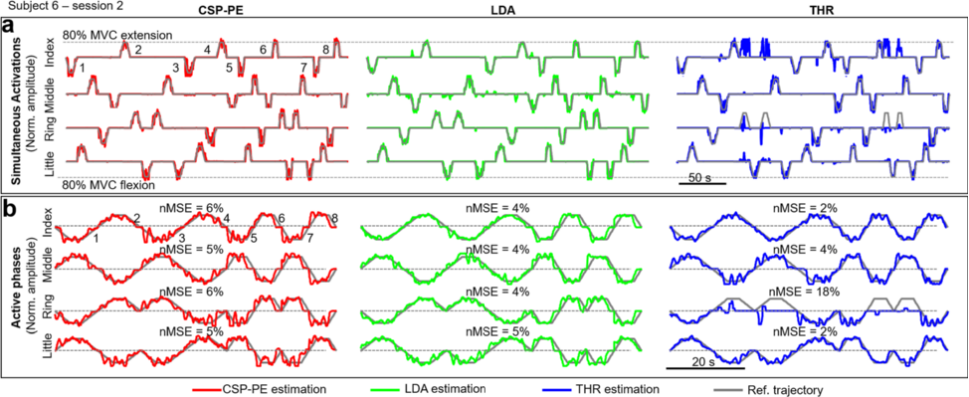
Representative results from one subject during online target task. The figure illustrates the quality of proportional tracking during the online test when using the three methods (CSP-PE, LDA and THR). The task for the subject was to activate the fingers, one at a time, tracking a reference trapezoidal trajectory. **a** The continuous colored and *gray lines* are the estimated and reference trajectories for each finger, including both silent (zero level) and active phases (trapezoids). **b** Active phases concatenated, with the indicated nMSE of estimation. With THR, false activations were more frequent and the ring extension could not be properly estimated

Figure 7 displays representative results from one subject illustrating the quality of proportional tracking when using the three methods. Figure 7-a depicts the reference and generated trajectories for each finger, including both silent and active sections, whereas Fig. 7-b zooms into the active phases only. The subject successfully tracked the reference trajectory during different segments (i.e., slopes and constant levels at 80 % MVC) and rates of change of the trapezoidal activation profiles. In the case of the CSP-PE and the LDA, the trajectory was well reconstructed during both flexion and extension movements. The control using the LDA resulted in more false activations compared to the CSP-PE (e.g., see index and middle fingers in Fig. 7-a), but the difference between the two methods was not so pronounced as in the offline experiments. The results for the thresholding (THR) were similar, as indicated by the outcome measures computed over the trial (Fig. 7-b), except for the ring finger for which the subject was unable to control the extension. The subject effort to activate the ring extension resulted in the false activation of the index flexion. The inability to control certain movements using the THR was also observed in other subjects; in the second session, three out of nine subjects were not able to activate a certain finger movement. This problem was not observed with the two machine learning approaches.

**Fig. 8:**
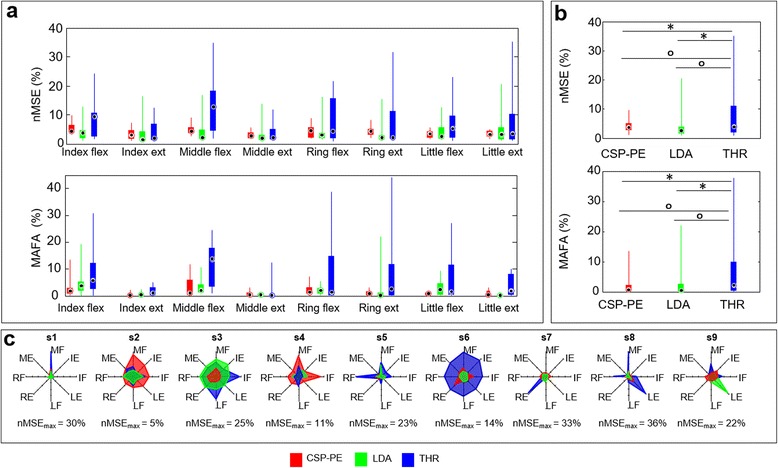
Summary results of the online experiment. **a**) Median and interquartile range (25–75 %) of the normalized mean square error between trapezoidal reference trajectory and control signals during active phases, and the mean amplitude of the false activations during silent phases across control methods for the eight finger movements. Overall, the online performance of the two machine learning methods was similar and better than the THR. **b**) Median and interquartile range (25–75 %) of the summary results shown in A. **c** Radar graph with the results for the accuracy of tracking (nMSE) for each subject. Each spoke represents one of the target finger movements and the length of the spokes is normalized to the maximum value of nMSE. The radar graphs for THR are characterized with spikes indicating larger errors, but the spikes are limited to some fingers (one, two typically), while for the other fingers the performance is actually comparable to that of the machine learning methods. *p < 0.01. The results of the distribution analysis are reported as circles ° *p* < 0.01

Group data are represented in Fig. 8-a, which shows the summary results across control methods for the eight finger movements during the online experiment. The statistical test showed that the finger movements and methods were significant factors as well as their interaction (*χ*^2^ = 43.52, *p* ≪0.01). There were no statistically significant differences between the methods for the same as well as across fingers. However, there was a trend indicating that the THR was the weakest control approach, i.e., the median nMSE as well as its IQR range were consistently higher compared to that of the LDA and the CSP-PE.

The results for the false activations complement those for the accuracy of tracking. The median MAFA was the highest for the THR consistently for all the movements, although there were no significant differences between the methods. However, some movements were controlled with substantially lower MAFA, for example, the MAFA for the index and middle finger extension was low with all three methods (e.g., compare to index flexion). Yet, the post hoc comparison did not reveal any significant differences between the specific methods across the movement classes.

The experiment was performed twice on consecutive days in order to evaluate the effect of training, and reducing the bias between subjects. Nevertheless, the lack of experience in EMG control may explain the overall variability between subjects showed in Fig. 8-c. For good control, the subject needs to activate each finger consistently, generating reproducible patterns of muscle activation that can be discriminated by the classifier/regressor. In addition, the patterns need to correspond to the ones generated during the training. The ability to reliably execute such patterns is likely subject dependent and two sessions were not enough for the consistency of control to improve and converge to a similar level across subjects. The figure shows the results for the accuracy of tracking (nMSE) for each subject as a radar graph, where each spoke represents one of the target finger movements and the length of the spokes was normalized to the maximum nMSE. This representation reveals participants who reached high level of accuracy with: i) all the three methods (s2 and s4), ii) the CSP-PE and the LDA (s1, s5, s6, s7 and s8), iii) the CSP-PE and the THR (s9) and finally iv) the CSP-PE only (s3). It is reasonable to expect that with practice, the online performance of the algorithms can be further improved, producing a more coherent behavior across participants [54]. The initial uncertainty and variability of control and substantial improvement due to training are characteristic for myoelectric control in general [55]. Regarding THR, for most subjects and movements the performance was similar to that of the CSP-PE and the LDA. However, as indicated by the few pronounced spikes in the radar plot, there were one or two specific movements in some subjects (see s1, s5, s7, and s8) that were substantially more difficult to control using THR. The performance dropped significantly in these few cases, decreasing the overall average accuracy and increasing the overall variability.

In the summary results shown in Fig. 8-b, the nMSE obtained with the CSP-PE, LDA and THR (median and IQR 25–75 %) was 3.6 % (2.5–5 %), 2.5 % (1.5–3.9 %) and 3.8 % (1.8–10.8 %), respectively. There was no statistically significant difference between the CSP-PE and the LDA, whereas the two machine learning methods were significantly different compared to the THR (*p* < 0.01). Furthermore, the CSP-PE and LDA exhibited similar dispersion, which was significantly lower compared to THR. The same trend holds for the MAFA. As shown in Fig. 8-b, the MAFA of the CSP-PE, LDA and THR was 0.7 % (0.2–2.2 %), 0.6 % (0.2–2.6) and 2.2 % (0.24–9.9 %), respectively. Both the CSP-PE and the LDA performed similarly, and they differed significantly with respect to THR (*p <* 0.01) both in median and dispersions.

The general similarity in the performance of the CSP-PE and the LDA was also confirmed by the CA (median and IQR 25–75 %), which were 91 % (87.7–91.7 %) for the CSP-PE and 90.3 % (89.4–93.25 %) for the LDA with no statistically significant differences.

## Discussion

The experiments demonstrated that the finger activation could be successfully decoded for different target activation profiles. Overall, the experiments demonstrated a more stable performance of the CSP-PE across the tested conditions. The CSP-PE exhibited less improvement between different sessions and outperformed the THR in online control and the LDA in offline tests. Furthermore, the study showed that a simple method, exploiting the topographical information about the individual finger activity from the HD-sEMG, provided in most cases regression accuracy similar to the pattern recognition techniques. However, THR lacked robustness in the sense that performance was not consistent across subjects and fingers.

### Offline and online proportional control

In the offline experiment, the performance of the CSP-PE and the LDA increased with the number of channels, saturating to a stable level for more than 24 electrodes. Importantly, for less than 24 electrodes, the CSP-PE outperformed the LDA consistently in all outcome measures, except CA, yielding more accurate force estimates in active phases and better suppression of false activations in the silent phases. This confirmed the hypothesis that the mathematical properties of the CSP-PE, as described in [8], make this method especially effective in the context of selective finger activation. Increasing the number of channels evened out the performance of the two methods with respect to the quality of tracking in the active phase, but did not change the superiority of the CSP-PE in filtering out the activations during the silent phases.

The results of the online experiments were in accordance with the insights from the offline tests. The tracking accuracy was similar with both the CSP-PE and the LDA, with the median nMSE of approximately 3.6 and 2.6 % in online experiment, and 3.6 and 3.5 % respectively in offline experiment (48 electrodes). As explained in Methods, the online experiment resulted in more than 24 electrodes selected by the experimenter. According to the trends revealed offline (Fig. 6), this number of channels was enough to even out the performances of the two algorithms. In addition, during online tests the subjects could use visual feedback to adapt the activations during the trial, as demonstrated in [40].

The CSP-PE exhibited more stable accuracy across the tested conditions. The performance was good from the beginning and similar across the two successive sessions, providing the least improvement in outcome measures. Furthermore, the CSP-PE produced better performance for lower number of channels (Fig. 6) compared to LDA. This means that the CSP-PE might be less sensitive to the subjective factors than the LDA, which produced similar accuracy but only for the high number of channels and after a session of practice.

### Electrode reduction

In the offline experiment, the electrodes were selected as a regular grid, without any relation to the specific finger activation patterns. Regular electrode grids are convenient for practical implementation and allow simple mounting, since they can be realized as extensible uniform bracelets that are simply wrapped around the forearm [56]. Future investigations will further address the minimization of the number of channels, determining acceptable electrode locations and optimizing electrode-recording configurations, using the established methods for feature reduction [57–59]. Importantly, the results of the present study (offline tests) demonstrate that the number of electrodes can be decreased substantially (from 192 to 10) without significantly compromising the performance. This is an optimistic result implying that the proposed methods could be translated into the clinical context using multichannel EMG braces comprising practical dry electrodes.

### Thresholding for online control

The online experiment demonstrated the feasibility of simple thresholding for proportional control of individual fingers, albeit with some limitations. Exploiting a dense array of detection points provided by HD-sEMG interface, distinct areas of focal sEMG activity could be identified for each finger [35], with an overlap in some cases due to anatomical constraints and crosstalk [37]. When the areas overlapped, the experimenter did not select the electrodes from the intersection. The THR method has a low computational cost and there is no training; after the electrode selection, the experimenter provided only a fast calibration of the thresholds associated with each finger movement. However, the THR method also exhibited some drawbacks. In the second session, three out of nine subjects were not able to activate a certain finger movement, due to a significant overlap, i.e., the activity of finger involved in the task projected strongly to neighboring areas triggering thereby other fingers. The THR was also less successful in suppressing the false activations. Nevertheless, when the subjects were able to control the finger, the tracking accuracy was actually comparable to the performances of the two machine learning approaches. Therefore, the THR is not universally applicable. However, it can be used successfully in some subjects or with a reduced number of movement classes, controlling only those fingers (or finger groups) characterized with distinct and separate areas of activity.

### Application for rehabilitation

The aforementioned conclusions could provide useful guidelines for the translation of the tested methods to the clinical context, targeting dexterous control of hand rehabilitation robots. For example, stroke patients have impaired motor functions characterized with pathological synergies. At the hand level, this is expressed as a difficulty in selectively activating individual fingers [34]. The methods developed in the present study could be applied to implement a user-driven control of a rehabilitation system. For example, the activation signals estimated for each finger, as demonstrated in the online tests, could be used as the control signals in the isometric mode of Amadeo, i.e., to implement the functionality of the force joystick, as when playing simple video games by producing appropriate isometric forces. More generally, the estimated signals could be used to trigger and/or modulate the movement of the Amadeo finger motors (dynamic control) [60]. Since the Amadeo system offers a set of therapeutic individual finger exercises, the future perspective is to integrate the individual finger myocontrol developed in the present study with these motivational tasks, creating thereby an innovative, engaging and user-responsive training program. Considering the future clinical application and following the results of the offline analysis, the control could be implemented with substantially less electrodes, which would allow using a practical EMG bracelet such as Myoband [56] combined with the CSP-PE. Alternatively, even a full HD-sEMG electrode system could be used practically if integrated in a textile garment (e.g., [37]). Importantly, for online control of Amadeo, the arm/hand will be supported exactly as in the present study, and it is therefore not required to train/test the algorithms with the arm in different positions, as usual in prosthetics to increase the robustness of the classification/regression across arm postures [61, 62].

Another potential application is the extension of the methods to the control of dexterous hand prostheses. At the current level, the methods tested in the present study could not be directly translated for general prosthetic application, as the fingers are controlled sequentially, one by one. However, such a controller could be used to support some specific functions (e.g., typing on a keyboard) exploiting the individual finger actuation available in the modern prostheses (e.g. i-Limb [63]). For example, keyboard typing could be implemented through classification (on-off), or the estimated force could be mapped to the finger velocity, allowing proportional control of the speed of finger flexion/extension (instead of force). The latter is not essential for typing, but it could allow the subject to type faster as he/she becomes more trained and skilled in control. More importantly, the present study demonstrates the feasibility of achieving fine and selective control of individual fingers, across a comprehensive number of tasks (force profiles) and with a reduced set of electrodes. Nevertheless, the control of a prosthesis requires a more natural and functional approach, and the future work will be to study the simultaneous regression of multiple fingers (including the thumb) using HD-sEMG setup.

The translation into the clinical context faces a number of challenges, which will be addressed in future work. For example, as explained above, stroke patients can have significant reduction in muscle forces and impaired coordinative control. Due to weak activity and pathological synergies [64], the activation patterns for the individual finger movements are likely to be significantly less discriminative and thereby more difficult to classify and estimate. In this context, nevertheless, an adaptive training can be envisioned in which the patient and the system coadapt [65] and evolve through the process of recovery. Initially, the system can estimate a subset of movements, limited to those that can be well discriminated. This can be used to start the training, promoting the initial recovery, and when the activity maps become better differentiated, new movements can be included.

Finally, as already pointed out in myoelectric control for prosthetics [66], it would be of interest for clinical applications to minimize the time and effort (subjects and staff) need for the training. Ideally, the training/calibration should be short and easy and without the need for frequent retraining. In the present study, we demonstrated that a reasonable quality of control can be achieved with an in-session training. The training is especially simple and easy to understand in the case of THR, since it reduces to selecting the channels with strong activation in a color map and then visually adjusting the thresholds (average time 10 min in THR vs. 20 min for LDA/CSP-PE). Therefore, the THR might also allow for an easy retraining across sessions. However, in the ideal case, the retraining would not be necessary. This was not tested in the present study but it is certainly an important future goal. The robustness of the methods in terms of retraining could be assessed by testing the control across sessions using the same, previously collected data (no retraining). In this context, the use of HD-sEMG interface might be particularly beneficial as an increased resolution contributes with redundant information, and this can be used to increase robustness. An illustrative demonstration is provided in [67] by extracting features reducing the sensitivity to electrodes shifts.

## Conclusion

The present study investigated methods for selective estimation of individual finger movements, motivated by the final aim of implementing an online protocol for dexterous finger control using a hand rehabilitation robot. We detected the intention to move a single finger from electromyographic signals providing proportional control while reducing the simultaneous co-activations of other fingers during both offline and online experiments. The insights from the present study can be used to guide the implementation of a practical myoelectric system for dexterous control in hand rehabilitation robotics and prosthetics. More specifically, the results demonstrated that despite the abundance of detection points in HD-sEMG, a simple method based on thresholding (THR) exhibited serious drawbacks, and that therefore the pattern recognition is still the method of choice for robust practical implementations. Next, provided that the recording is above a certain resolution (>24 channels), either of the pattern recognition methods (CSP-PE and LDA) can be selected to implement the control. In this case, information redundancy compensates for the favorable mathematical properties of the CSP-PE vs. LDA. Finally, if only a reduced number of electrodes is available (≤12), the CPS-PE is the recommended approach.

## Abbreviations

CA, classification accuracy; CSP-PE, common spatial pattern proportional estimator; DIP, distal interphalangeal; HD-sEMG, high density surface electromyography; IE, index extension; IF, index flexion; IQR, interquartile range; LDA, linear discriminant analysis; LE, little extension; LF, little flexion; MAFA, mean amplitude of the false activations; MCP, metacarpophalangeal; ME, middle extension; MF, middle flexion; MVC, maximum voluntary contraction; nMSE, normalized mean square error; PCORR, Pearson correlation coefficient; PIP, proximal interphalangeal; RE, ring extension; RF, ring flexion; RMS, root mean square; THR, thresholding

## References

[1] Schieber MH, Poliakov AV (1998). Partial inactivation of the primary motor cortex hand area: effects on individuated finger movements. J Neurosci.

[2] Oby ER, Ethier C, Miller LE (2013). Movement representation in the primary motor cortex and its contribution to generalizable EMG predictions. J Neurophysiol.

[3] Asghari Oskoei M, Hu H (2007). Myoelectric control systems—a survey biomed. Signal Process Control.

[4] Fougner A, Stavdahl O, Kyberd PJ, Losier YG, Parker PA (2012). Control of upper limb prostheses: terminology and proportional myoelectric control-a review. IEEE Trans Neural Syst Rehabil Eng.

[5] Nielsen JLG, Holmgaard S, Jiang N, Englehart KB, Farina D, Parker PA (2011). Simultaneous and proportional force estimation for multifunction myoelectric prostheses using mirrored bilateral training. IEEE Trans Biomed Eng.

[6] Muceli S, Member S, Farina D, Member S (2012). Simultaneous and proportional estimation of hand kinematics from EMG during mirrored movements at multiple degrees-of-freedom. IEEE Trans Neural Syst Rehabil Eng.

[7] Jiang N, Englehart KB, Parker PA (2009). Extracting simultaneous and proportional neural control information for multiple-DOF prostheses from the surface electromyographic signal. IEEE Trans Biomed Eng.

[8] Amsuess S, Gobel P, Graimann B, Farina D (2014). A multi-class proportional myocontrol algorithm for upper limb prosthesis control: validation in real-life scenarios on amputees. IEEE Trans neural Syst Rehabil Eng.

[9] Cipriani C, Zaccone F, Micera S, Carrozza MC (2008). On the shared control of an EMG-controlled prosthetic hand: analysis of user–prosthesis interaction. IEEE Trans Robot.

[10] Belter JT, Segil JL, Dollar AM, Weir RF (2013). Mechanical design and performance specifications of anthropomorphic prosthetic hands: A review. J Rehabil Res Dev.

[11] Chiri A, Vitiello N, Giovacchini F, Roccella S, Vecchi F, Carrozza MC (2012). Mechatronic design and characterization of the index finger module of a hand exoskeleton for post-stroke rehabilitation. IEEE/ASME Trans Mechatronics.

[12] Tong KY, Member S, Hu XL, Fung KL, Wei XJ, Rong W, Susanto EA (2011). An EMG-driven exoskeleton hand robotic training device on chronic stroke subjects task training system for stroke rehabilitation. IEEE Int Conf Rehabil Robot.

[13] Wege A, Kondak K, Hommel G. Force control strategy for a hand exoskeleton based on sliding mode position control. IEEE/RSJ Int Conf Intell Robot Syst. 2006:4615–4620.

[14] Zhang X, Zhou P (2012). High-density myoelectric pattern recognition. IEEE Trans Biomed Eng.

[15] Al-Timemy AH, Bugmann G, Escudero J, Outram N (2013). Classification of finger movements for the dexterous hand prosthesis control with surface electromyography. IEEE J Biomed Heal Informatics.

[16] Merletti R, Parker PA. Electromyography: Physiology, Engineering, and Non-Invasive Applications. 2004.

[17] Shrirao N a, Reddy NP, Kosuri DR (2009). Neural network committees for finger joint angle estimation from surface EMG signals. Biomed Eng Online.

[18] Antfolk C, Cipriani C, Controzzi M, Carrozza MC, Sebelius F. Using EMG for real-time prediction of joint angles to control a prosthetic hand equipped with a sensory feedback system. J Med Biol Eng. 2010, 30:399–406.

[19] Hioki M, Kawasaki H. Estimation of Finger Joint Angles from sEMG Using a Neural. ISRN Rehabil. 2012, 2012.

[20] Ngeo JG, Tamei T, Shibata T (2014). Continuous and simultaneous estimation of finger kinematics using inputs from an EMG-to-muscle activation model. J Neuroeng Rehabil.

[21] Castellini C, Koiva R. Using surface electromyography to predict single finger forces. 2012 4th IEEE RAS EMBS Int. Conf. Biomed. Robot. Biomechatronics. 2012:1266–1272.

[22] Gijsberts A, Bohra R, Sierra González D, Werner A, Nowak M, Caputo B, Roa M a, Castellini C (2014). Stable myoelectric control of a hand prosthesis using non-linear incremental learning. Front Neurorobot.

[23] Ravindra V, Castellini C (2014). A comparative analysis of three Non-invasive human-machine interfaces for the disabled. Front Neurorobot.

[24] Taub E, Uswatte G, Elbert T (2002). New treatments in neurorehabilitation founded on basic research. Nat Rev Neurosci.

[25] Biitefisch C, Hummelsheim H, Denzler P (1995). Repetitive training of isolated movements improves the outcome of motor rehabilitation of the centrally paretic hand. J Neurol Sci.

[26] Langhorne P, Bernhardt J, Kwakkel G (2011). Stroke rehabilitation. Lancet.

[27] Johansson BB (2011). Current trends in stroke rehabilitation. A review with focus on brain plasticity. Acta Neurol Scand.

[28] Merians AS, Poizner H, Boian R, Burdea G, Adamovich S (2006). Sensorimotor training in a virtual reality environment: does it improve functional recovery poststroke? Neurorehabil. Neural Repair.

[29] Norouzi-Gheidari N, Archambault PS, Fung J (2012). Effects of robot-assisted therapy on stroke rehabilitation in upper limbs: systematic review and meta-analysis of the literature. J Rehabil Res Dev.

[30] Colombo R, Pisano F, Micera S, Mazzone A, Delconte C, Carrozza MC, Dario P, Minuco G (2005). Robotic techniques for upper limb evaluation and rehabilitation of stroke patients. IEEE Trans Neural Syst Rehabil Eng.

[31] Mulas M, Folgheraiter M, Gini G. An EMG-Controlled Exoskeleton for Hand Rehabilitation. 9th Int. Conf. Rehabil. Robot. 2005. ICORR 2005. 2005:371–374.

[32] Dipietro L, Ferraro M, Palazzolo JJ, Krebs HI, Volpe BT, Hogan N (2005). Customized interactive robotic treatment for stroke: EMG-triggered therapy. IEEE Trans Neural Syst Rehabil Eng.

[33] Prange GB, Jannink MJ a, Groothuis-Oudshoorn CGM, Hermens HJ, IJzerman MJ (2006). Systematic review of the effect of robot-aided therapy on recovery of the hemiparetic arm after stroke. J Rehabil Res Dev.

[34] Schieber MH, Lang CE, Reilly KT, McNulty P, Sirigu A (2009). Selective activation of human finger muscles after stroke or amputation. Adv Exp Med Biol.

[35] Leijnse JN a L, Campbell-Kyureghyan NH, Spektor D, Quesada PM (2008). Assessment of individual finger muscle activity in the extensor digitorum communis by surface EMG. J Neurophysiol.

[36] Gallina A, Botter A (2013). Spatial localization of electromyographic amplitude distributions associated to the activation of dorsal forearm muscles. Front Physiol.

[37] Gazzoni M, Celadon N, Mastrapasqua D, Paleari M, Margaria V, Ariano P (2014). Quantifying forearm muscle activity during wrist and finger movements by means of multi-channel electromyography. PLoS One.

[38] Peleg D, Braiman E, Yom-Tov E, Inbar GF (2002). Classification of finger activation for use in a robotic prosthesis arm. IEEE Trans Neural Syst Rehabil Eng.

[39] Tenore FVG, Ramos A, Fahmy A, Acharya S, Etienne-Cummings R, Thakor NV (2009). Decoding of individuated finger movements using surface electromyography. IEEE Trans Biomed Eng.

[40] Jiang N, Rehbaum H, Vujaklija I, Graimann B, Farina D. Intuitive, online, simultaneous, and proportional myoelectric control over two degrees-of-freedom in upper limb amputees. IEEE Trans Neural Syst Rehabil Eng. 2014;22:501–10. 10.1109/TNSRE.2013.2278411 23996582

[41] Gijsberts A, Atzori M, Castellini C, Muller H, Caputo B (2014). Movement error rate for evaluation of machine learning methods for sEMG-based hand movement classification. IEEE Trans Neural Syst Rehabil Eng.

[42] Birdwell JA, Hargrove LJ, Weir RF, Kuiken T a (2015). Extrinsic finger and thumb muscles command a virtual hand to allow individual finger and grasp control. IEEE Trans Biomed Eng.

[43] Englehart K, Hudgins B (2003). A robust, real-time control scheme for multifunction myoelectric control. IEEE Trans Biomed Eng.

[44] Englehart K, Hudgins B, Parker PA, Member S (2001). A wavelet-based continuous classification scheme for multifunction myoelectric control. IEEE Trans Biomed Eng.

[45] Fukunaga K. Introduction to Statistical Pattern Recognition. 2013.

[46] Scheme EJ, Member S, Englehart KB, Member S, Hudgins BS, Member S (2011). Selective classification for improved robustness of myoelectric control under nonideal conditions. IEEE Trans Biomed Eng.

[47] Parker P, Englehart K, Hudgins B (2006). Myoelectric signal processing for control of powered limb prostheses. J Electromyogr Kinesiol.

[48] Hahne JM, Graimann B, Muller KR (2012). Spatial filtering for robust myoelectric control. IEEE Trans Biomed Eng.

[49] Tomioka R, Lemm S (2008). Filters for Robust EEG. IEEE Signal Process Mag.

[50] Ramoser H, Müller-Gerking J, Pfurtscheller G (2000). Optimal spatial filtering of single trial EEG during imagined hand movement. IEEE Trans Rehabil Eng.

[51] Blankertz B, Dornhege G, Krauledat M, Müller K-R, Curio G (2007). The non-invasive Berlin Brain-Computer Interface: fast acquisition of effective performance in untrained subjects. Neuroimage.

[52] Lorrain T, Jiang N, Farina D (2011). Influence of the training set on the accuracy of surface EMG classification in dynamic contractions for the control of multifunction prostheses. J Neuroeng Rehabil.

[53] Lang CE (2004). Human finger independence: limitations due to passive mechanical coupling versus active neuromuscular control. J Neurophysiol.

[54] Jiang N, Vujaklija I, Rehbaum H, Member S, Graimann B (2014). Is accurate mapping of EMG signals on kinematics needed for precise online myoelectric control ?. IEEE Trans Neural Syst Rehabil Eng.

[55] Radhakrishnan SM, Baker SN, Jackson A (2008). Learning a novel myoelectric-controlled interface task. J Neurophysiol.

[56] Myo armband. [https://www.thalmic.com/]. Accessed 12 May 2016.

[57] Geng Y, Zhang X, Zhang Y-T, Li G (2014). A novel channel selection method for multiple motion classification using high-density electromyography. Biomed Eng Online.

[58] Huang H, Zhou P, Li G, Kuiken T (2009). Spatial filtering improves EMG classification accuracy following targeted muscle reinnervation. Ann Biomed Eng.

[59] Hwang HJ, Hahne JM, Müller KR (2014). Channel selection for simultaneous myoelectric prosthesis control. 2014 Int. Winter Work. Brain-Computer Interface. BCI.

[60] “Amadeo solution”, Tyromotion GmbH [http://tyromotion.com/wp-content/uploads/2013/04/HartwigM-2014-The-Tyrosolution-Concept._EN.pdf]. Accessed 12 May 2016.

[61] Geng Y, Zhou P, Li G. Toward attenuating the impact of arm positions on electromyography pattern-recognition based motion classification in transradial amputees. J Neuroeng Rehabil. 2012;9(74):1–11. 10.1186/1743-0003-9-74 PMC3551659 23036049

[62] Fougner A, Scheme E, Member S, Chan ADC, Member S, Englehart K, Member S, Stavdahl Ø (2011). Resolving the limb position effect in myoelectric pattern recognition. IEEE Trans Neural Syst Rehabil Eng.

[63] “i-Limb Ultra: User Manual” Touch Bionics Ltd. [http://www.touchbionics.com/sites/default/files/files/i-limb ultra revolution user manual September 2014.pdf]. Accessed 12 May 2016.

[64] Li X, Liu J, Li S, Wang Y (2014). Examination of hand muscle activation and motor unit indices derived from surface EMG in chronic stroke. IEEE Trans Biomed Eng.

[65] Blank AA, French JA, Pehlivan AU, O’Malley MK (2014). Current trends in robot-assisted upper-limb stroke rehabilitation: promoting patient engagement in therapy. Curr Phys Med Rehabil Reports.

[66] Farina D, Member S, Jiang N, Rehbaum H, Member S, Holobar A, Graimann B, Dietl H, Aszmann OC (2014). The extraction of neural information from the surface EMG for the control of upper-limb prostheses: emerging avenues and challenges. IEEE Trans Neural Syst Rehabil Eng.

[67] Stango A, Member S, Negro F, Farina D, Member S (2015). Spatial correlation of high density EMG signals provides features robust to electrode number and shift in pattern recognition for myocontrol. IEEE Trans Neural Syst Rehabil Eng.

[68] You K-J, Rhee K-W, Shin H-C (2010). Finger motion decoding using EMG signals corresponding various arm postures. Exp Neurobiol.

[69] Cipriani C, Antfolk C, Controzzi M, Lundborg G, Rosen B, Carrozza MC, Sebelius F (2011). Online myoelectric control of a dexterous hand prosthesis by transradial amputees. IEEE Trans Neural Syst Rehabil Eng.

[70] Khushaba RN, Kodagoda S, Takruri M, Dissanayake G (2012). Toward improved control of prosthetic fingers using surface electromyogram (EMG) signals. Expert Syst Appl.

[71] Sebelius F, Eriksson L, Balkenius C, Laurell T (2006). Myoelectric control of a computer animated hand: a new concept based on the combined use of a tree-structured artificial neural network and a data glove. J Med Eng Technol.

